# Relationships between physical activity and quality of life in parent–child dyads with ASD

**DOI:** 10.3389/fpsyg.2025.1669728

**Published:** 2025-10-20

**Authors:** Yu Song, Bo Shen, Yanli Pang, Liangshan Dong

**Affiliations:** ^1^Jimei University, Xiamen, China; ^2^Division of Kinesiology, Wayne State University, Detroit, MI, United States; ^3^Department of Physical Education, Central China Normal University, Wuhan, China; ^4^China University of Geoscience, Wuhan, China

**Keywords:** physical activity, quality of life, parent–child dyads, family context, relationships

## Abstract

**Introduction:**

Autism spectrum disorder (ASD) is characterized by core and associated symptoms that adversely affect the quality of life (QOL) of both children with ASD and their parents. Although physical activity (PA) has been shown to promote QOL and well-being, limited research has examined these associations within parent–child dyads in families affected by ASD.

**Methods:**

This cross-sectional study recruited 85 parent–child dyads from two autism rehabilitation centers in Central China. Children had a mean age of 5.25 years, and 75.3% of parents were aged between 31 and 40 years. Partial Pearson correlation analyses were conducted to examine associations between children’s and parents’ PA levels and multiple domains of QOL, controlling for child age, sex, and symptom severity.

**Results:**

Significant reciprocal associations were observed between the PA levels of children with ASD and their parents. Specifically, children’s light-intensity physical activity (LPA) was positively associated with parents’ LPA (*r* = 0.351, *p* < 0.01) and with the psychological (*r* = 0.23, *p* < 0.05) and environmental (*r* = 0.26, *p* < 0.05) domains of parental QOL. No direct correlations were identified between parental PA and children’s QOL.

**Discussion:**

These findings underscore the potential of LPA as a feasible and accessible form of joint activity that may support QOL within families of children with ASD. Framed through reciprocal determinism, the results highlight the interconnected roles of children’s PA (behavior), parents’ psychological well-being (personal factor), and the family context (environment). Further longitudinal and intervention studies are warranted to confirm these relationships and inform family-centered PA interventions.

## Introduction

Autism spectrum disorder (ASD) is a neurodevelopmental condition characterized by persistent challenges in social communication and interaction, restricted interests, and repetitive behaviors (American Psychiatric Association, 2013). It frequently coexists with various developmental challenges, including intellectual disability, attention-deficit/hyperactivity disorder, behavioral disorders, language delays, motor impairments, epilepsy, sleep disturbances, and feeding difficulties (Lord et al., 2020). These lifelong symptoms can substantially affect the quality of life (QOL) of both children with ASD and their parents. Quality of life, a multidimensional concept, encompasses well-being and health across physical, emotional, and social domains (Evers et al., 2022). Since the initial QOL study in 2003 on individuals with autism, a growing body of research has demonstrated that autism’s core and co-occurring symptoms can adversely affect at least one QOL domain in children with ASD (Delahaye et al., 2014; Kuhlthau et al., 2013; Menezes and Mazurek, 2021; Potvin et al., 2015).

Raising a child with ASD places a substantial burden on parents and families. A recent nationwide survey reported that families affected by ASD face considerable costs for medical and nonmedical care, along with reduced parental productivity (Zhao et al., 2023). Approximately 85% of individuals with autism are unable to live independently, which means lifelong care often falls on parents or family members (Mailick Seltzer et al., 2001). These parents must devote extra emotional energy and time to supporting their children and adjusting their daily lives. Extensive research indicates that parents of children with ASD report lower QOL than parents of typically developing children (Vasilopoulou and Nisbet, 2016), particularly in physical and mental health domains (Benson and Karlof, 2009; Estes et al., 2009; Karst and Van Hecke, 2012). Thus, improving the QOL of both children with ASD and their parents is an important area of concern.

A growing body of research has investigated the association between QOL and physical activity (PA). Evidence suggests that regular PA enhances QOL across age groups—including children, adolescents, adults, and the elderly—and reduces the risk of various diseases (Okely et al., 2021). The World Health Organization (WHO) recommends regular PA for children with disabilities to support both physical and mental aspects of QOL (Carty et al., 2021; Okely et al., 2021). However, children with ASD (Jones et al., 2017; Liang et al., 2020; Rech et al., 2022) and parents of children with disabilities (Ku and Sung, 2022) tend to engage in PA less frequently. For children with ASD, barriers such as motor challenges, social difficulties, and lack of environmental support can significantly hinder PA participation (Arkesteyn et al., 2022; Liang et al., 2020). Parents also face barriers, including time constraints (Rizk et al., 2011) and physical discomfort (Miodrag et al., 2015; Miodrag and Hodapp, 2010). Given these challenges, it remains unclear whether positive associations between PA and QOL apply equally to children with ASD and their parents.

For example, Neville et al. (2021) examined the link between PA participation and adaptive behavior related to QOL in children with ASD (mean age = 32 ± 4 months). Their study found a positive association between PA and both physical and social aspects of QOL. Building on this, two large-scale studies investigated the effects of 24-h activity guidelines on health-related QOL in children and adolescents with autism (ages 6–17). In a database-driven study, Kong et al. (2023) reported that individuals meeting only the PA recommendations had fewer challenges in adaptive skills, such as dressing and bathing. Similarly, a cross-national study by Li et al. (2022) found that children adhering to recommended PA guidelines generally reported higher QOL.

Correlation studies also support this association in mothers of children with autism. Bourke-Taylor et al. (2012) observed a strong positive relationship between QOL and maintaining a healthy lifestyle—spending time outside the home and engaging in regular PA—in mothers of children with ASD aged 5–18. More recently, Tsunoda et al. (2023) confirmed that physical exercise positively influences psychological well-being in mothers of children with autism (mean age = 11.3).

While these studies support the positive link between PA and QOL, prior research has mainly focused on school-aged children, adolescents, or parents in isolation. Few studies have examined preschool-aged children with ASD and their parent–child dyads simultaneously, despite the preschool years being a critical period for both children and parents. This period provides a unique opportunity for parents to enhance family QOL through targeted training and support, fostering greater engagement in play, daily routines, and PA (Nevill et al., 2018). By focusing on preschool-aged children and their parent–child dyads, the present study addresses an underexplored population and extends previous research beyond older children and adolescents.

The reciprocal relationship between parents and children during the preschool years can be conceptualized through Bandura’s theory of reciprocal determinism, particularly in the context of family health promotion (Glanz et al., 2008). According to this theory, individuals possess distinct qualities—such as thoughts, motivations, and behaviors—that continuously influence one another over time. Within a family, each member shapes and is shaped by the shared environment. This bidirectional interaction suggests that both individual behaviors and the family context are mutually influential. Although reciprocal determinism has been applied in health psychology research, its use in studies of PA and QOL in ASD contexts remains limited. By explicitly applying this framework, the present study goes beyond simple correlational analyses, providing a theoretically grounded interpretation of the feedback loops between parents’ and children’s PA behaviors and family QOL.

This study aims to examine the relationship between PA and QOL in parent–child dyads with autism during the preschool stage, focusing on the family context. Previous studies have largely focused on moderate-to-vigorous physical activity (MVPA) in relation to QOL; therefore, this study also assesses the impact of light-intensity PA and total PA participation. The novelty of this study lies in its dual focus: (1) investigating preschool-aged children with ASD and their parents—an understudied group in PA–QOL research—and (2) integrating Bandura’s reciprocal determinism to interpret bidirectional influences within parent–child dyads. Guided by existing research and reciprocal determinism theory, the following hypotheses were proposed: (1) PA levels in parent–child dyads are positively associated, (2) PA levels in children with ASD are positively associated with their parents’ QOL, and (3) PA levels in parents are positively associated with their children’s QOL.

## Methods

### Participants

This study specifically focused on preschool-aged children with ASD and their parents, as this developmental stage represents a critical period for early intervention and family adaptation. A total of 94 parent–child dyads were recruited from two autism rehabilitation institutions in central China. The inclusion criteria were as follows: (1) Children must have a confirmed clinical diagnosis of ASD. The Chinese version of the Autism Diagnostic Interview-Revised (ADI-R), adapted from Berument et al. (1999), was used for assessment. For children aged 4 years and older, a cut-off score of 15 on the Social Communication Questionnaire (SCQ) within the ADI-R indicated susceptibility to ASD. For children aged 2–4 years, a cut-off score of 11 was applied due to lower test sensitivity in this age range (Wiggins et al., 2007). (2) Children were required to be between 3 and 6 years of age. This preschool age range was deliberately chosen because it represents a key stage for structured rehabilitation and family-based support, and because the collaborating rehabilitation centers primarily serve children within this age bracket, making the sampling decision both theoretically and practically justified. (3) The child’s father or mother had to serve as the primary caregiver. The final sample included 85 parent–child dyads; participants’ demographics are shown in Table 1.

**Table 1:** Participants’ demographics (*N* = 85).

Characteristic	# of participants (SD/%)
ASD	
Age	5.25 (SD = 1.48)
Sex	
Males	73 (85.90%)
Females	12 (14.10%)
Number of siblings	
Only child	59 (62.80%)
1 sibling	30 (31.90%)
2 siblings	5 (5.30%)
Educational placement	
Not integrated	64 (75.30%)
Partially integrated	16 (18.80%)
Fully integrated	5 (5.90%)
Parents	
Caregiver roles	
Mother	73 (85.90%)
Father	12 (14.10%)
Age	
26 ~ 30 yr	12 (14.10%)
31 ~ 35 yr	42 (49.40%)
36 ~ 40 yr	22 (25.90%)
41 ~ 60 yr	9 (10.60%)
Employment status	
Full-time employed	18 (21.20%)
Part-time employed	5 (5.90%)
Full-time caregiver	62 (72.90%)

### Procedure

All participants were briefed on the study and provided informed consent prior to participation. They were informed of their right to withdraw at any time if they experienced discomfort. Data were collected across three time points: January, March, and July 2022. All families met the inclusion criteria, and duplicate participation was avoided.

### Measures

#### Participant demographic

Parents reported demographic information including their child’s age, sex, and symptom severity. These variables were considered key factors potentially influencing the QOL of both children and their parents, as identified in previous studies (Chen et al., 2024; Chuang et al., 2014; Kuhlthau et al., 2018; Limbers et al., 2009; Tung et al., 2014).

#### Symptom severity

Children’s symptom severity was assessed using the Social Communication Questionnaire (SCQ), current edition (Berument et al., 1999). This 40-item instrument evaluates ASD symptoms with binary “yes” or “no” responses. Except for the first item assessing phrase-level speech, each item is scored as 1 for abnormal behavior and 0 for typical behavior. Total scores range from 0 to 39, with higher scores indicating greater symptom severity. The Chinese version of the SCQ has been validated as a reliable and accurate tool for assessing ASD symptoms in Chinese-speaking populations (Gau et al., 2011). Previous studies have employed the SCQ to evaluate symptom severity in children with ASD (Song et al., 2022). In the present study, Cronbach’s alpha ranged from 0.73 to 0.88, indicating acceptable internal consistency.

### Quality of life

Children’s QOL was measured using the Pediatric Quality of Life Inventory, Version 4.0 (PedsQoL) (Varni et al., 2003). The PedsQoL is a 23-item scale scored on a five-point Likert scale, with a 21-item version available for children aged 2–4. The scale provides both parent/caregiver proxy-reported and child self-reported versions, evaluating children’s QOL across physical, emotional, social, and school domains. Given the communication challenges of children with ASD, parent proxy-reports were used. Parents rated their child’s behavioral challenges over the past month on a scale from “never problems” to “often problems.” Ratings were converted into numerical scores from 0 to 100, with higher scores indicating better QOL. The Chinese PedsQoL has demonstrated satisfactory psychometric properties (Hao et al., 2010). In this study, Cronbach’s alpha ranged from 0.77 to 0.89, reflecting good internal consistency.

Parents’ QOL was assessed using the Chinese version of the World Health Organization Quality of Life-BREF (WHOQOL-BREF), a widely validated cross-cultural instrument. The WHOQOL-BREF includes 26 items, with two items focusing specifically on overall well-being and physical health. Scores across four domains—physical, psychological, social relationships, and environmental factors—were standardized on a 0–100 scale for consistency (Skevington et al., 2004). This tool has been previously applied to parents of children with ASD (Wainer et al., 2017). In the current study, Cronbach’s alpha ranged from 0.85 to 0.92, indicating strong internal consistency.

### Physical activity

PA levels of children with ASD and their parents were measured using the Godin Leisure-Time Exercise Questionnaire (GLTEQ) (Godin, 2011), which estimates weekly PA in metabolic equivalents (MET). Parents recalled the frequency of their own and their child’s engagement in different PA intensities over the past week. GLTEQ scores were calculated in two steps: first, frequency of light, moderate, and vigorous PA was recorded; then, these frequencies were multiplied by 3, 5, and 9, respectively, to reflect energy expenditure. Total weekly scores were obtained by summing these products: Weekly leisure-time activity score = (9 × vigorous) + (5 × moderate) + (3 × light). Scores were categorized into light-intensity PA (LPA), moderate-to-vigorous PA (MVPA), and total PA for comparison across studies. The GLTEQ has been widely used in multiple populations, including children with ASD through parent proxy-reporting (Esentürk and Yarımkaya, 2021; Healy and Marchand, 2020).

### Data collection and analysis

Statistical analyses were performed using IBM SPSS version 26.0. The Kolmogorov–Smirnov test confirmed that all variables were continuous and approximately normally distributed, with skewness and kurtosis ranging from −0.49 to 1.26 and −0.79 to 1.53, respectively (George and Mallery, 2016). To examine associations between PA and QOL in parent–child dyads, partial Pearson correlations were conducted controlling for symptom severity, age, and sex. A significance threshold of *p* < 0.05 was applied. For interpretive clarity, correlation coefficients were categorized as follows: 0.25 indicating weak, 0.49 moderate, and 0.69 strong correlations (Cohen, 2013).

## Results

Partial Pearson correlation analyses were conducted to examine associations between different levels of PA in children with ASD and their parents (see Table 1). The results revealed a moderate positive correlation between LPA scores in children and those in their parents (*r* = 0.351, *p* < 0.01). Additionally, a weak positive correlation was observed between children’s total GLTEQ scores and their parents’ MVPA scores (*r* = 0.255, *p* < 0.05) as well as total GLTEQ scores (*r* = 0.279, *p* < 0.05).

Further partial correlation analyses were conducted to explore associations between PA and multiple domains of QOL in both children with ASD and their parents (see Tables 2, 3). Notably, children’s LPA scores were weakly but positively correlated with parents’ psychological QOL (*r* = 0.23, *p* < 0.05) and environmental QOL (*r* = 0.26, *p* < 0.05). No significant correlations were found between parents’ PA levels and children’s QOL scores (Table 4).

**Table 2:** The association between PA in children with ASD and their parents.

	1	2	3	4	5	6
1. C_MVPA	1					
2. C_LPA	−0.274*	1				
3. C_GLTQE	0.862**	0.252*	1			
4. P_MVPA	0.172	0.155	0.255*	1		
5. P_LPA	−0.017	0.351**	0.167	0.266*	1	
6. P_GLTQE	0.168	0.208	0.279*	0.968**	0.477**	1

C_MVPA, children with ASD’s weekly moderate and vigorous physical activity score; C_LPA, children with ASD’s weekly light-intensity physical activity score; C_GLTQE, children with ASD’s weekly total PA score; P_MVPA, parents’ weekly moderate and vigorous physical activity score; P_LPA, parents’ weekly light-intensity physical activity score; P_GLTQE, parents’ weekly total PA score.

^*^Significant at the 0.05 level; ^**^Significant at the 0.01 level.

**Table 3:** The association between PA in children with ASD and QOL in their parents.

	1	2	3	4	5	6	7	8	9
1. C_MVPA	1								
2. C_LPA	−0.274*	1							
3. C_GLTQE	0.862**	0.252*	1						
4. Overall well-being	−0.06	0.178	0.033	1					
5. Overall physical health	−0.144	0.149	−0.066	0.786**	1				
6. Physical	−0.053	0.073	−0.015	0.658**	0.674**	1			
7. Psychological	−0.052	0.225*	0.066	0.733**	0.642**	0.770**	1		
8. Social relationship	0.014	0.002	0.015	0.718**	0.704**	0.717**	0.771**	1	
9. Environmental	0.021	0.229*	0.142	0.576**	0.555**	0.721**	0.740**	0.642**	1

C_MVPA, children with ASD’s weekly moderate and vigorous physical activity score; C_LPA, children with ASD’s weekly light-intensity physical activity score; C_GLTQE, children with ASD’s weekly total PA score.

^*^Significant at the 0.05 level; ^**^Significant at the 0.01 level.

**Table 4:** The association between PA in parents and QOL in their children.

	1	3	3	4	5	6	7
1. P_MVPA	1						
2. P_LPA	0.266*	1					
3. P_GLTQE	0.968**	0.477**	1				
4. Physical	0.155	−0.008	0.16	1			
5. Emotional	0.053	−0.033	0.036	0.157	1		
6. Social	−0.051	0.18	−0.002	0.137	0.198	1	
7. School	0.077	0.034	0.088	0.276*	0.277*	0.215	1

P_MVPA, parents’ weekly moderate and vigorous physical activity score; P_LPA, parents’ weekly light-intensity physical activity score; P_GLTQE, parents’ weekly total PA score.

^*^Significant at the 0.05 level; ^**^Significant at the 0.01 level.

## Discussion

Previous research has examined the relationship between PA and QOL in adolescents with ASD and their parents. However, most studies have focused primarily on moderate-to-vigorous physical activity (MVPA), with limited attention to preschool-aged children. This gap highlights the need to investigate potential associations between PA and QOL in preschool-aged children with ASD and their parent–child dyads. Given the high level of interaction between preschool-aged children and their parents, it is essential to consider the reciprocal influences of PA and QOL within the family context. The primary aim of this study was to examine associations, rather than causal links, between PA and QOL in preschool-aged children with ASD and their parents.

Drawing on the concept of reciprocal determinism, this study first examined associations between PA engagement in children with ASD and their parents. Results revealed a significant positive correlation between LPA levels in children and their parents. Additionally, children’s total PA was positively associated with parents’ MVPA and total PA. Recent research has emphasized the role of parental support in promoting PA participation in children with ASD (Brown et al., 2020; Lu et al., 2023). However, few studies have directly examined bidirectional associations between child and parent PA. For example, Ayvazoglu et al. (2015) reported no significant correlations in a small sample, although trends suggested consistent PA patterns in younger families. The present study extends these findings by including preschool-aged children, providing additional statistical evidence for the association. Despite the statistical significance of the moderate correlation between child and parent LPA (*r* = 0.351), effect sizes are small-to-moderate and should be interpreted cautiously. We hypothesize that this association may reflect the developmental needs of preschool children, who often require active parental involvement during physical activities.

Although no direct correlation was observed between parental PA involvement and the QOL of children with ASD, the present study identified a favorable association between children’s LPA and the psychological domain of parental QOL. This pattern is consistent with evidence from studies in typical families (Benton et al., 2015; Duarte et al., 2012; Loprinzi, 2015; Reiner et al., 2015) and single-parent families (Maher et al., 2018), which have reported links between children’s PA and mothers’ psychological well-being. Our findings also partially align with a recent study by Sahan et al. (2022), who demonstrated a positive association between improvements in children’s physical fitness and enhancements in the psychological QOL of mothers of children with ASD. While their study directly assessed physical fitness, our results suggest a complementary perspective, as physical fitness can be conceptualized as a long-term outcome of sustained PA. Taken together, these findings indicate that parents’ psychological well-being may play an important mediating role in this relationship.

Nevertheless, due to the cross-sectional design, the associations identified here should not be interpreted as causal. Emerging evidence shows that regular PA can improve both physical and mental health in children with ASD (Brown et al., 2020). Such improvements may reshape parental perceptions of their child’s impairment, thereby enhancing their psychological QOL (Hayes and Watson, 2013; Mello et al., 2019). This interplay reflects the principle of reciprocal determinism, in which children’s PA (behavior) and parents’ psychological well-being (personal factor) interact within the shared family context (environment).

Interestingly, LPA—but not other forms of PA—was positively associated with parental psychological QOL. Preschool-aged children with ASD often require more emotional support and guidance from parents during physical activities, making LPA an accessible form for joint engagement. Previous research has shown that parental involvement in interventions enhances parents’ psychological well-being (Wainer et al., 2017), and PA participation can strengthen perceptions of parental competence. These findings illustrate feedback loops in Bandura’s reciprocal determinism model: parents’ involvement shapes children’s engagement, while children’s responses influence parental psychological outcomes, all within a shared environment. Such interactions may be more pronounced in families with ASD than in typical households, highlighting the unique dynamics of PA in these families.

Moreover, children’s LPA was positively associated with the environmental domain of parental QOL. This association may reflect shared participation in LPA activities within the family. Children’s engagement in activities such as outdoor play, walking, or using exercise equipment can enhance parents’ perceptions of environmental support, contributing to higher satisfaction in this domain. Barriers to PA for children with ASD include inadequate infrastructure, limited facility accessibility, and low community awareness (Arnell et al., 2020; Nichols et al., 2019; Obrusnikova and Cavalier, 2011; Obrusnikova and Miccinello, 2012). Access to parks, green spaces, and inclusive facilities, combined with community awareness, shapes families’ likelihood of engaging in PA. Reciprocal determinism further explains this pattern: the environment both constrains and facilitates activity and is simultaneously influenced by parents’ perceptions and engagement. These findings underscore the importance of fostering inclusive and accessible environments to promote joint PA, benefiting both children with ASD and their families.

### Limitations

This study has several limitations that should be acknowledged. First, the generalizability of the findings may be limited by the specific cultural and socioeconomic context, as the sample was drawn exclusively from families attending two special education schools in Central China. Cultural and institutional factors, such as the availability of inclusive environments and access to healthcare, may further constrain the applicability of the findings to other regions or countries. Second, the relatively small sample size necessitates further validation through large-scale surveys to ensure the robustness of the results.

Third, while PA recall questionnaires are efficient and sensitive in capturing exercise-related activity, they may lack precision. Specifically, the Godin Leisure-Time Exercise Questionnaire (GLTEQ), although previously applied in ASD populations, relies on self- or proxy-report. This approach is vulnerable to recall bias and social desirability effects, and may not fully capture the nuances of children’s PA patterns. Future studies should incorporate more objective measures, such as accelerometers, along with activity logs, to provide unbiased and detailed assessments.

Fourth, the study did not collect detailed socioeconomic or educational information from parents, both of which are known to influence PA participation and QOL. The absence of these variables limits the ability to account for potentially important confounding factors. Fifth, the majority of respondents were mothers, which may bias the findings toward maternal perceptions of child QOL and underrepresent fathers’ experiences.

Finally, while the results suggest that engaging in joint physical activities may predict and improve QOL in families with autism during the preschool years, understanding of these shared behaviors remains speculative. Future research should incorporate detailed behavioral logs to document and analyze joint activities more comprehensively. This approach could provide valuable insights for interventions aimed at enhancing family dynamics and well-being.

### Implications

Our findings highlight a reciprocal relationship between PA and QOL, showing that children’s PA levels are positively associated with the psychological and environmental well-being of their parents. However, the effect sizes observed were generally small to moderate and should be interpreted with caution. These results underscore the potential for parents and children to engage in light-intensity physical activity (LPA) together during the preschool years, providing shared opportunities that may support QOL for both parties. Importantly, this emphasis on LPA does not diminish the critical role of MVPA in promoting broader physical and mental health.

These findings emphasize the value of a family-centered approach grounded in reciprocal determinism, where children’s PA (behavior), parents’ psychological well-being (personal factor), and the shared family environment (context) mutually influence one another. Future interventions should prioritize integrated programs that actively involve both children with ASD and their parents. Such programs could include parent training to equip caregivers with the skills needed to guide children’s movement and activity participation, while simultaneously enhancing overall PA levels and QOL for the parent–child dyads.

Evidence from prior research supports the efficacy of collaborative PA and shared learning experiences (Columna et al., 2024; Johnson et al., 2022). By leveraging these reciprocal processes, integrated approaches have the potential to strengthen family cohesion, promote well-being, and offer sustainable, context-sensitive strategies for health promotion in households with children with ASD.

## Data Availability

The original contributions presented in the study are included in the article/supplementary material, further inquiries can be directed to the corresponding author.
